# Supplementary material for: Inferring protein expression changes from mRNA in Alzheimer’s dementia using deep neural networks
Source: Nat Commun. 2022 Feb 3;13:655. doi: 10.1038/s41467-022-28280-1 (PMC8814036; doi:10.1038/s41467-022-28280-1)
Supplement: Supplementary file 1 — Supplementary Information [file 41467_2022_28280_MOESM1_ESM.pdf]

Supplemental Information

	All cases	TMT cases	SRM cases	RNA cases	PCG cases	AC cases
N	1192	384	629	808	645	705
Age at death (mean (sd))	89.56 (6.53)	89.24 (6.48)	89.61 (6.53)	89.72 (6.56)	89.56 (6.53)	89.30 (6.49)
Female (n, %)	808 (67.8)	266 (69.3)	421 (66.9)	542 (67.1)	406 (62.9)	458 (65.0)
Years of education (mean, sd)	16.20 (3.54)	15.84 (3.59)	16.34 (3.57)	16.37 (3.50)	16.24 (3.44)	16.35 (3.58)
AD (n, %)	428 (35.9)	103 (26.8)	257 (40.9)	325 (40.2)	207 (32.1)	227 (32.2)
Global AD pathology (mean, sd)	0.73 (0.63)	0.65 (0.57)	0.79 (0.65)	0.78 (0.65)	0.70 (0.61)	0.73 (0.63)
Amyloid score (mean, sd)	4.25 (4.18)	4.78 (4.70)	4.32 (4.08)	3.99 (3.89)	3.93 (3.96)	4.30 (4.27)
Tangles score (mean, sd)	7.03 (8.56)	4.79 (4.98)	7.86 (9.21)	8.10 (9.64)	6.45 (8.50)	6.56 (8.23)

Supplementary Table 1. Demographic information for the ROSMAP cohort.

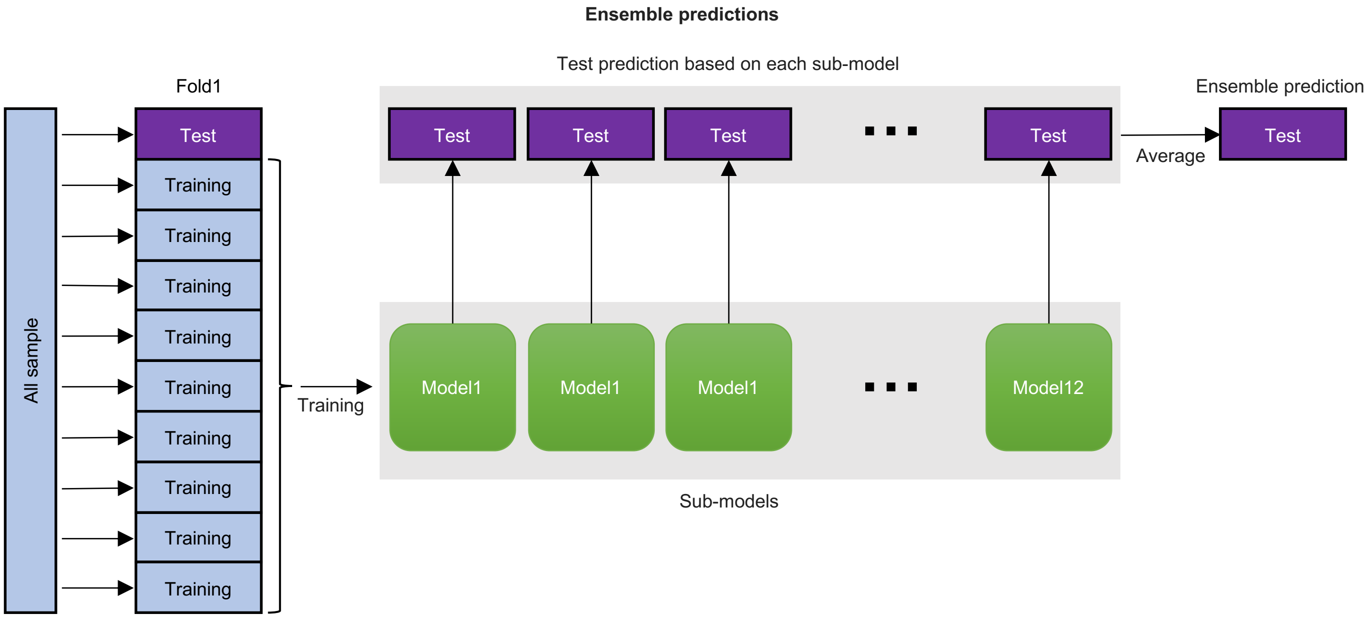

Figure S1. The procedure of ensemble prediction. We split 384 individuals into 10 groups and then took a group as a holdout test set. Using data from the remaining groups of individuals, we trained 12 clei2block models. Each model was trained with a different combination of input variables but the same individuals. Each of the trained models was applied to the test set, which resulted in generating 12 predictions for protein levels. Finally, we take an average of the 12 predictions to obtain a consensus prediction.

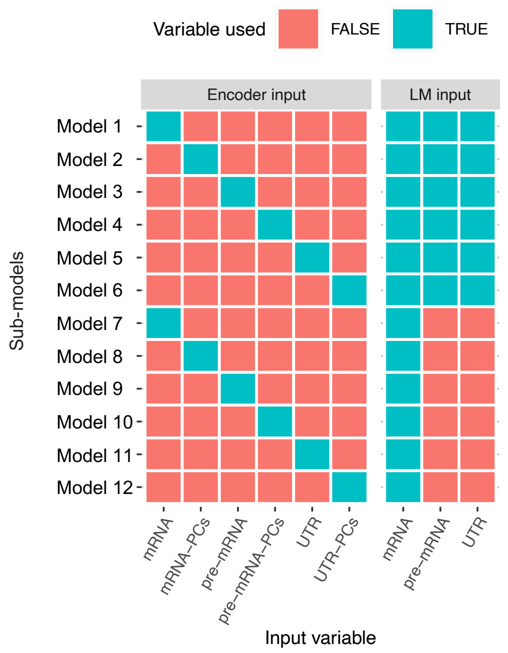

Figure S2. The input variables used for each sub-model.

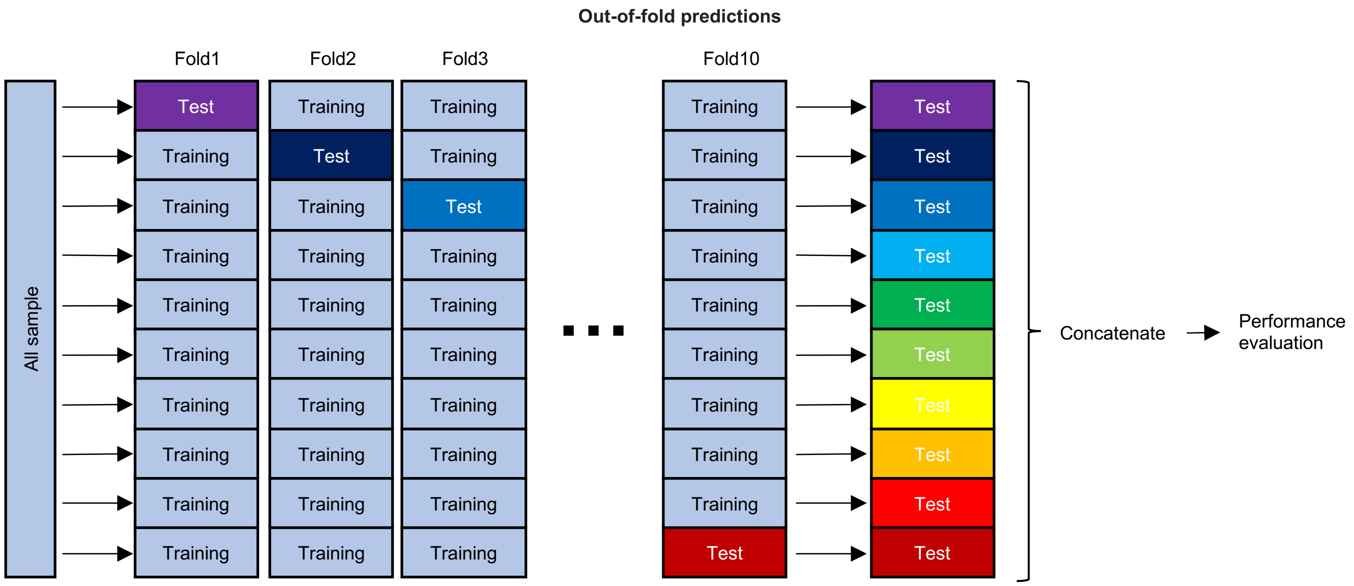

Figure S3. The procedure of out-of-fold predictions. We split 384 individuals into 10 groups and then took a group as a holdout test set. Using data from the remaining groups of individuals, we trained the ensemble models and generated a consensus prediction for the test set. By repeating this process for all 10 folds, we obtained out-of-fold predictions for all 384 samples. Finally, we compared the predicted protein levels with the actual protein levels to evaluate the performance of the model.

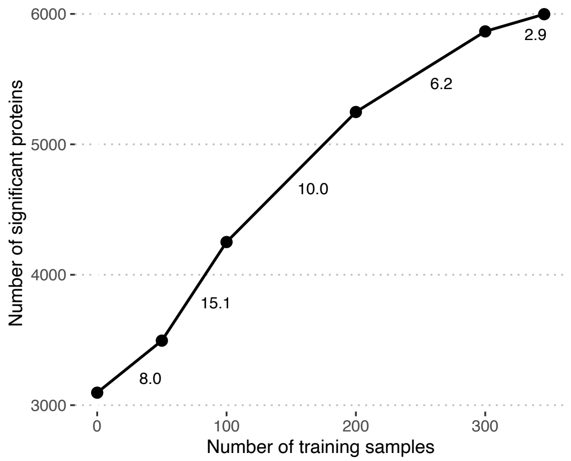

Figure S4. Relationship between sample size and model performance. The clei2block models were trained with reduced training sets (n=50, 100, 200, 300, and 345). The trained models were applied to the hold-out samples to compute the model accuracy as described in the method. The baseline performance (n=0) corresponds to the number of proteins correlated with corresponding mRNAs.

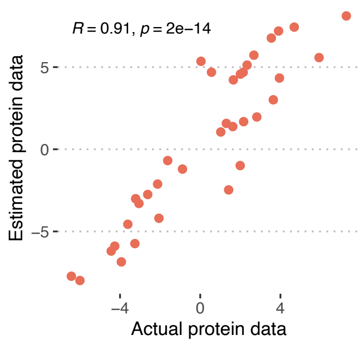

Figure S5. Comparison of disease association of modules between actual and estimated proteomes. Module expression levels based on the actual proteome (n=384) and the predicted proteomes from the RNA cohort (n=808) were contrasted between AD and controls with accounting for the age of death, sex, and the length of education. The scatter plot indicates a relationship between t-statistics from the two separate data. Pearson's correlation test was used for this comparison.

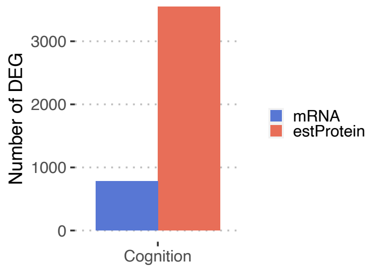

Figure S6. The number of genes associated with cognitive score. The estimated proteomes and transcriptomes from the same individual (n=808) were tested for their associations with cognitive scores accounted for the age of death, education, and sex. A Bonferroni-corrected p-value less than 0.05 was set as a significance threshold.

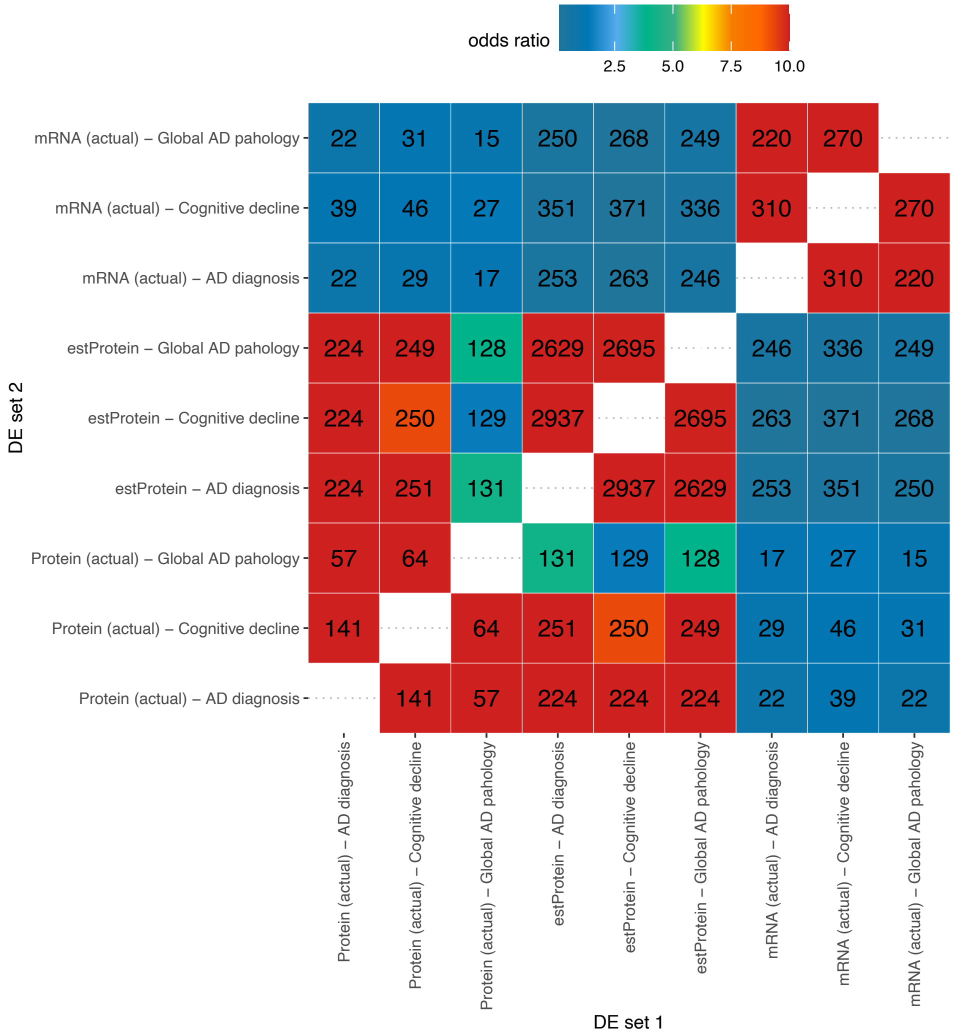

Figure S7. Comparison of trait-associated genes between data sets and traits. Differentially expressed genes from each transcriptomic or proteomic data were compared using Fisher's exact test. The color of each panel corresponds to the odds ratio of co-occurrence of differentially expressed genes. The number of common genes is indicated in each panel.

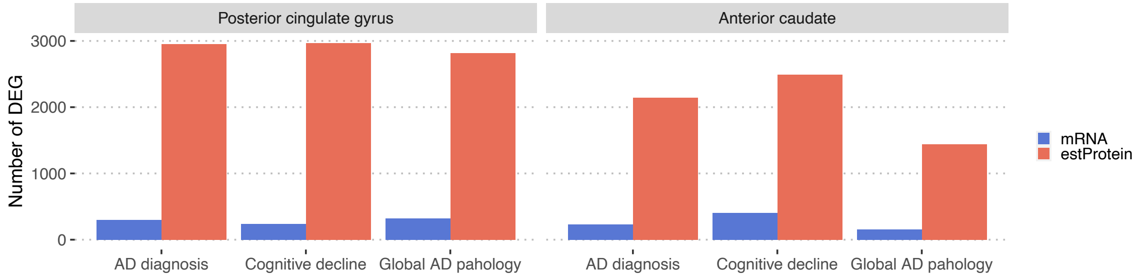

Figure S8. The number of genes associated with ADRD-phenotypes in PCG and AC regions. The estimated proteomes and transcriptomes from PCG (n=705) and AC (n=645) were tested for their associations with cognitive decline, AD diagnosis, and global AD pathology. A Bonferroni-corrected p-value less than 0.05 was set as a significance threshold.

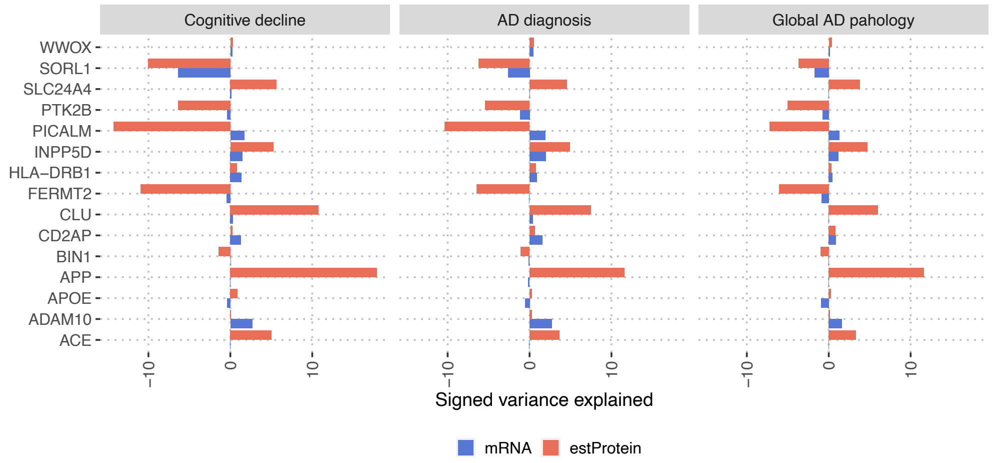

Figure S9. Variance of AD GWAS genes explained by AD-phenotypes.

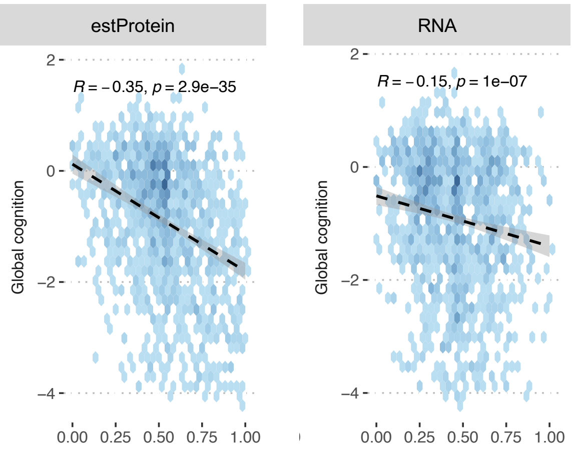

Figure S10. Association of pseudotime with cognitive score. We used Pearson's correlation for this association test. The shaded areas represent the 95% confidence level interval for predictions from a linear model.

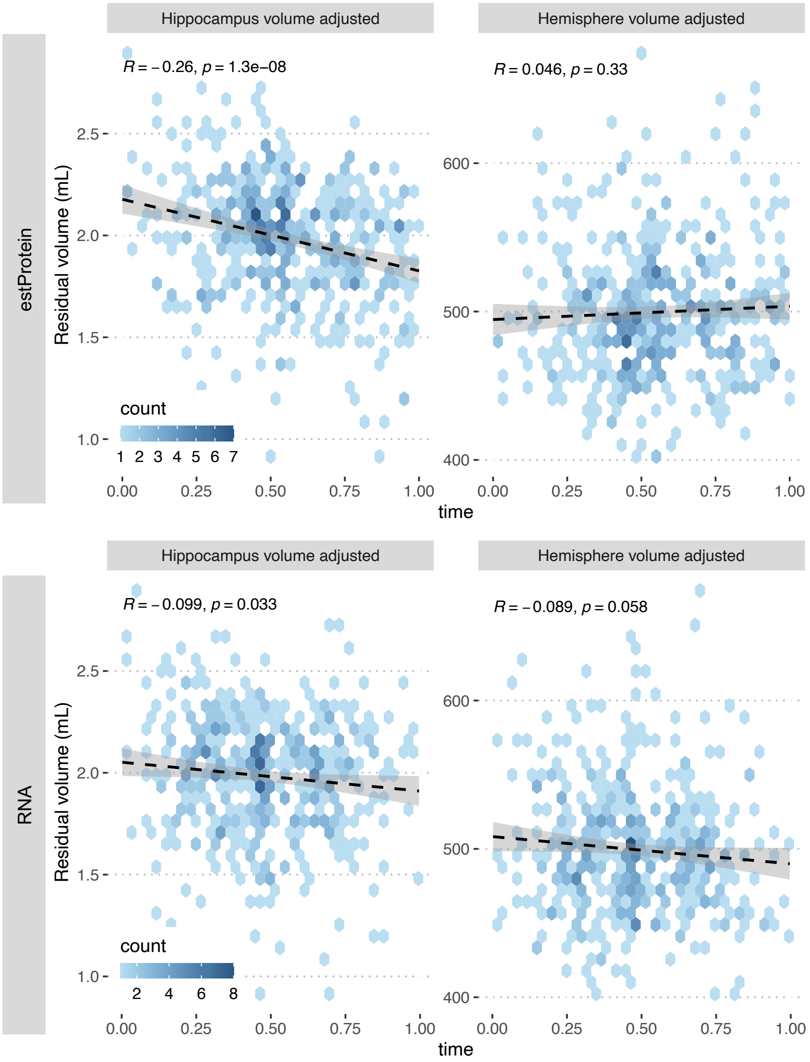

Figure S11. Protein pseudotime in DLPFC is correlated with hippocampal volume normalized to hemisphere volume. To normalize hippocampal volume with respect to hemisphere volume, we regressed hippocampal volume with hemisphere volume to obtain residual hemisphere volume. We also normalized hemisphere volume with respect to hippocampal volume. The plots show the relationships of these residuals and the protein pseudotime in DLPFC. We used Pearson's correlation for this association test. The shaded areas represent the 95% confidence level interval for predictions from a linear model.

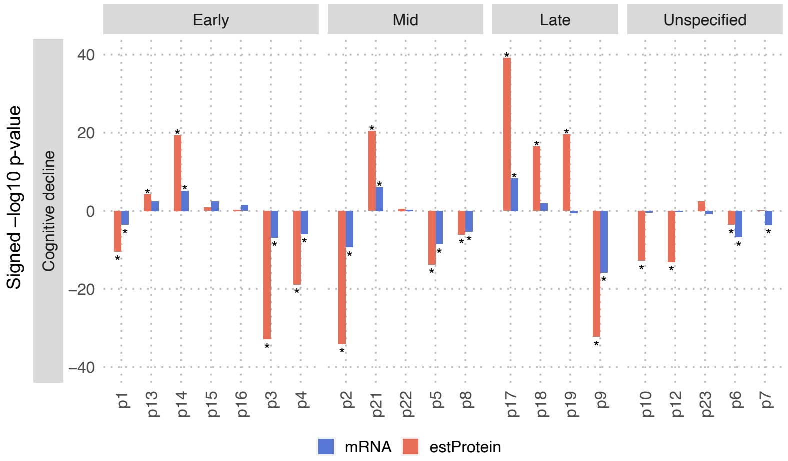

Figure S12. Associations of protein modules with cognitive decline. Module expression levels based on the predicted proteomes from the DLPFC (n=1,192) were regressed with cognitive decline and the age of death. For reference, we also computed module expression levels of RNA expression given module definition based on the predicted proteomes. Negative log10 of the p-value with the sign of its association to cognitive decline is indicated in the bar plot. We used limma for this test. The asterisk represents a Bonferroni-corrected p <0.05.

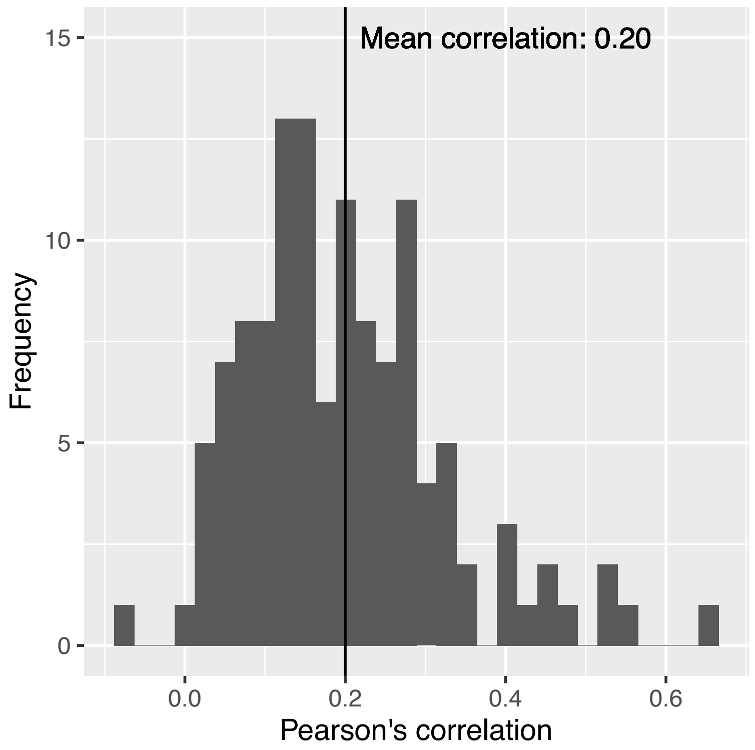

Figure S13. Correlation of protein abundance measured by tandem mass tag (TMT) system and selected reaction monitoring (SRM). We quantified the abundance of 121 proteins in the mid-frontal region of 384 subjects using two independent technologies: TMT proteomics and SRM proteomics. The histogram represents the distribution of Pearson’s correlation between the protein abundance measured by TMT and SRM.

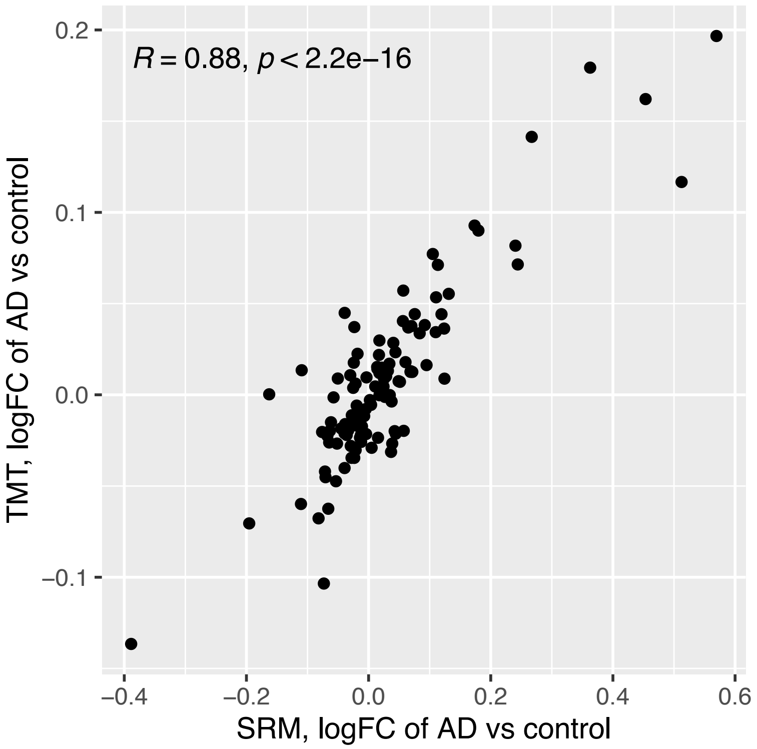

Figure S14. Comparison of TMT and SRM datasets in log-fold change between AD and control. We used Pearson's correlation for this comparison.

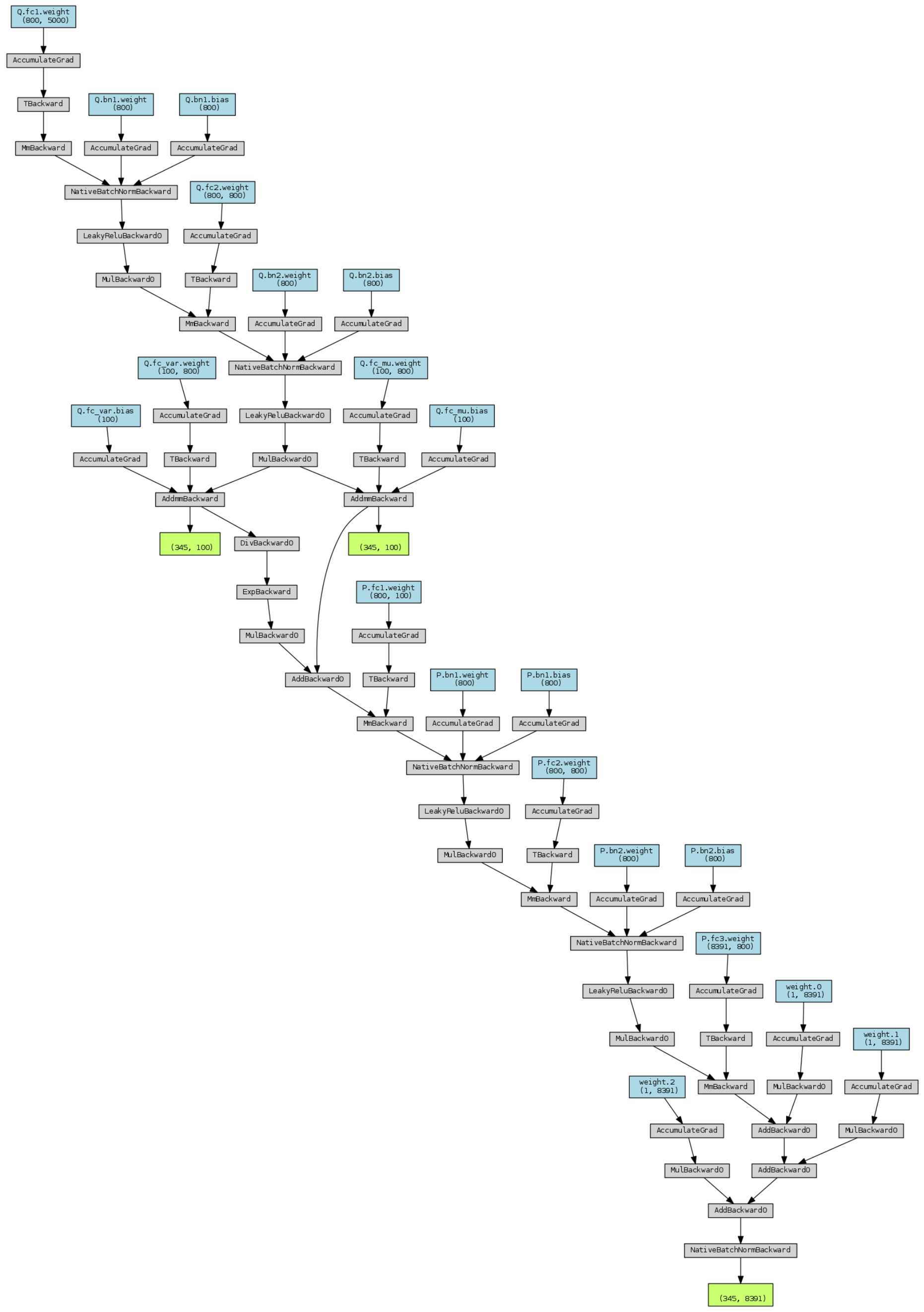

Figure S15. The detailed model architecture of clei2block.
