# Supplementary material for: Inferring protein expression changes from mRNA in Alzheimer’s dementia using deep neural networks
Source: Nat Commun. 2022 Feb 3;13:655. doi: 10.1038/s41467-022-28280-1 (PMC8814036; doi:10.1038/s41467-022-28280-1)
Supplement: Supplementary file 2 — Reporting Summary [file 41467_2022_28280_MOESM2_ESM.pdf]

nature research

Corresponding author(s): Shinya Tasaki

Last updated by author(s): Nov 11, 2021

Reporting Summary

Nature Research wishes to improve the reproducibility of the work that we publish. This form provides structure for consistency and transparency in reporting. For further information on Nature Research policies, see our Editorial Policies and the Editorial Policy Checklist.

Statistics

For all statistical analyses, confirm that the following items are present in the figure legend, table legend, main text, or Methods section.

n/a	Confirmed	
☐	☒	The exact sample size (*n*) for each experimental group/condition, given as a discrete number and unit of measurement
☐	☒	A statement on whether measurements were taken from distinct samples or whether the same sample was measured repeatedly
☐	☒	The statistical test(s) used AND whether they are one- or two-sided<br>*Only common tests should be described solely by name; describe more complex techniques in the Methods section.*
☐	☒	A description of all covariates tested
☐	☒	A description of any assumptions or corrections, such as tests of normality and adjustment for multiple comparisons
☐	☒	A full description of the statistical parameters including central tendency (e.g. means) or other basic estimates (e.g. regression coefficient) AND variation (e.g. standard deviation) or associated estimates of uncertainty (e.g. confidence intervals)
☐	☒	For null hypothesis testing, the test statistic (e.g. *F*, *t*, *r*) with confidence intervals, effect sizes, degrees of freedom and *P* value noted<br>*Give P values as exact values whenever suitable.*
☒	☐	For Bayesian analysis, information on the choice of priors and Markov chain Monte Carlo settings
☒	☐	For hierarchical and complex designs, identification of the appropriate level for tests and full reporting of outcomes
☐	☒	Estimates of effect sizes (e.g. Cohen's *d*, Pearson's *r*), indicating how they were calculated

Our web collection on statistics for biologists contains articles on many of the points above.

Software and code

Policy information about availability of computer code

Data collection	We used publicly available software, as described in the methods, to pre-process the RNA sequence data and extract features.

Data analysis	WGCNA 1.69, https://cran.r-project.org/web/packages/WGCNA<br>limma 3.42.2, https://bioconductor.org/packages/release/bioc/html/limma.html<br>SpeakEasy 1.0, http://www.cs.rpi.edu/~szymansk/SpeakEasy/index.html<br>SCORPIUS 1.0.7, https://cran.r-project.org/web/packages/SCORPIUS<br>GradientExplainer 0.35.0, https://shap-lrjball.readthedocs.io/en/latest/index.html<br>glmnet 4.1, https://cran.r-project.org/web/packages/glmnet<br>STAR 2.6, https://github.com/alexdobin/STAR<br>Picard tools 2.23.1, https://broadinstitute.github.io/picard/<br>Kallisto 0.46, https://pachterlab.github.io/kallisto/<br>CQN 1.3.2, https://bioconductor.org/packages/release/bioc/html/cqn.html<br>QAPA 1.3.1, https://github.com/morrislab/qapa<br>Pytorch 1.2, https://pytorch.org<br>CatBoost 0.21, https://catboost.ai<br>igraph 1.2.6, https://igraph.org<br>scikit-learn 0.24.2, https://scikit-learn.org/<br>relaimpo 2.2-5, https://cran.r-project.org/web/packages/relaimpo/index.html<br>Automatic Registration Toolbox released on 9/22/2011, https://www.nitrc.org/frs/?group_id=90<br>The deep-neural protein translation model is available at https://github.com/stasaki/clei2block.<br>The code for trajectory analysis is available at https://github.com/stasaki/SCORPIUS.

For manuscripts utilizing custom algorithms or software that are central to the research but not yet described in published literature, software must be made available to editors and reviewers. We strongly encourage code deposition in a community repository (e.g. GitHub). See the Nature Research guidelines for submitting code & software for further information.

Data

Policy information about availability of data

All manuscripts must include a data availability statement. This statement should provide the following information, where applicable:

- Accession codes, unique identifiers, or web links for publicly available datasets
- A list of figures that have associated raw data
- A description of any restrictions on data availability

Data used in these analyses, the predicted proteome data, and the estimated pseudotimes can be requested at the RADC Resource Sharing Hub at www.radc.rush.edu or downloaded from the Synapse repository (http://dx.doi.org/10.7303/syn3219045, http://dx.doi.org/10.7303/syn3159438). The deep-neural protein translation model is available at https://github.com/stasaki/clei2block and http://dx.doi.org/10.7303/syn23624037. The code for trajectory analysis is available at https://github.com/stasaki/SCORPIUS. The RNA-seq and protein data are available via the AD Knowledge Portal (https://adknowledgeportal.org). The AD Knowledge Portal is a platform for accessing data, analyses, and tools generated by the Accelerating Medicines Partnership (AMP-AD) Target Discovery Program and other National Institute on Aging (NIA)-supported programs to enable open-science practices and accelerate translational learning. The data, analyses, and tools are shared early in the research cycle without a publication embargo on secondary use. Data is available for general research use according to the following requirements for data access and data attribution (https://adknowledgeportal.org/DataAccess/Instructions).

Field-specific reporting

Please select the one below that is the best fit for your research. If you are not sure, read the appropriate sections before making your selection.

☒ Life sciences ☐ Behavioural & social sciences ☐ Ecological, evolutionary & environmental sciences

For a reference copy of the document with all sections, see nature.com/documents/nr-reporting-summary-flat.pdf

Life sciences study design

All studies must disclose on these points even when the disclosure is negative.

Sample size	We used all samples for which RNA-seq, proteomics, and phenotype data are available. These samples represent all subjects with available frozen brain samples at the time of data generation.
Data exclusions	All data meeting pre-determined quality control criteria were included for analysis.
Replication	For each experiment, we have at least 196 biological replicates and all attempts at replication successfully support the conclusions of this study.
Randomization	There is no randomization. ROS and MAP are community studies. We enroll everyone who doesn't have dementia at the time of enrollment and agrees to annual detailed clinical and cognitive evaluation. All participants with an available sample were used in the analyses.
Blinding	The team members generating the data from the biological samples were blinded to the phenotypic data of each participant. The data analyst is required to access to the molecular and phenotypic data in order to extract information necessary for the planned analysis, execute quality control of the data and preprocessing, integrate the data with other data resources, and conduct statistical tests.

Reporting for specific materials, systems and methods

We require information from authors about some types of materials, experimental systems and methods used in many studies. Here, indicate whether each material, system or method listed is relevant to your study. If you are not sure if a list item applies to your research, read the appropriate section before selecting a response.

Materials & experimental systems

n/a	Involved in the study
☒	☐ Antibodies
☒	☐ Eukaryotic cell lines
☒	☐ Palaeontology and archaeology
☒	☐ Animals and other organisms
☐	☒ Human research participants
☒	☐ Clinical data
☒	☐ Dual use research of concern

Methods

n/a	Involved in the study
☒	☐ ChIP-seq
☒	☐ Flow cytometry
☐	☒ MRI-based neuroimaging

Human research participants

Policy information about studies involving human research participants

Population characteristics	All human subjects are participants in one of two prospective studies of aging (ROS and MAP). ROS/MAP are community-based cohort studies. As community-based cohorts, ROS/MAP have much less referral bias, which can introduce significant sociodemographic, clinical, and genetic differences into studies of patients. At enrollment, mean education was 16.2 years, 67.8% were female, 98.4% were non-Latino white, and 23.2% had one or more APOE 4 alleles. While all were without known dementia at study enrollment, 7.8% met a research diagnosis for dementia at their baseline evaluation. These are community based observational studies. They are not our patients. Information on other diagnoses and treatments are limited. The mean age at death was 89.6 years and 35.9% were diagnosed with Alzheimer's disease at the time of death.
Recruitment	The ROS study is comprised of older catholic priests, nuns, and monks throughout the USA. The MAP study recruits older lay persons from the greater Chicago area. Participants are not compensated for their participations. All visits and data collection other than optional biennial MRI are done as home visits to ensure convenience for the participant and data close to death.
Ethics oversight	The ROS and MAP studies were each approved by an Institutional Review Board (IRB) of Rush University Medical Center. Both studies enroll older persons without known dementia. All participants agree to an annual detailed clinical evaluation and organ donation at the time of death. Prior to enrollment, each participant signed an informed consent and an Anatomical Gift Act for (AGA) donation of brain, spinal cord, nerve, and muscle to the investigators for research purposes. The AGA is recognized in all 50 states and the District of Columbia. It is an advanced directive that foregoes the need to obtain consent for autopsy from a next of kin at the time of death. Participants also sign a repository consent to allow their data and biospecimens to be shared in accordance with procedures established by the relevant IRB.

Note that full information on the approval of the study protocol must also be provided in the manuscript.

Magnetic resonance imaging

Experimental design

Design type	Ex vivo brain MRI
Design specifications	At autopsy brains were hemisected in accordance with standard protocol and the cerebral hemisphere with more visible pathology was immersed in 4% paraformaldehyde solution and refrigerated at 4°C. At approximately one-month postmortem the hemisphere from each decedent was imaged using a 3-Tesla MRI scanner after allowing the tissue to warm to room temperature.
Behavioral performance measures	This study does not contain behavioral performance measures.

Acquisition

Imaging type(s)	Structural image (T2-weighted)
Field strength	3-Tesla
Sequence & imaging parameters	Fast spin-echo T2-weighted MRI data with at least two different echo times: Field of View (FOV) 160mm*160mm; Acquisition matrix 256mm*256mm; slice thickness 1.5mm; repetition time (TR) 3600~4055 ms; scan time 31-35 min.
Area of acquisition	Hemisected brain

Diffusion MRI ☐ Used ☒ Not used

Preprocessing

Preprocessing software	Automatic Registration Toolbox (ART: Ardekani et al. 2005)
Normalization	Using ART, the S0 volume from each brain hemisphere was warped to match the template. The deformation field map defining this transformation was then applied to that same hemisphere's T2 weighted volume.
Normalization template	A study-specific template was created from 30 subjects (30 hemispheres).
Noise and artifact removal	This study did not perform noise and artifact removal.
Volume censoring	This study did not perform volume censoring.

Statistical modeling & inference

Model type and settings	This study did not directly incorporate MRI images as predictors or outcomes in the statistical modeling; instead, hippocampal volume was extracted and used as a scalar variable in the modeling.
Effect(s) tested	This study did not test effects utilizing MRI images directly, as described above.

Specify type of analysis: ☐ Whole brain ☒ ROI-based ☐ Both

Anatomical location(s)	Images were warped to a previously developed postmortem cerebral hemisphere template, on which we had manually drawn a mask encompassing the hippocampus. We back-transformed this mask onto images in their original space by applying the inverse of the individual-to-template transform to the mask image, as previously described. After eliminating non-tissue-containing voxels, we extracted the resultant volume of each back-transformed mask, yielding a measure of hippocampal volume. The total hemisphere volume was calculated based on the number of tissue-containing voxels.
Statistic type for inference (See Eklund et al. 2016)	As described above, this study did not directly incorporate MRI images as predictors or outcomes in the statistical modeling; instead, hippocampal volume was extracted and used as a scalar variable in the modeling.
Correction	This study did not test effects utilizing MRI images directly, so no correction was applied.

Models & analysis

n/a	Involved in the study
☒	☐ Functional and/or effective connectivity
☒	☐ Graph analysis
☒	☐ Multivariate modeling or predictive analysis
