# Supplementary material for: Inferring protein expression changes from mRNA in Alzheimer’s dementia using deep neural networks
Source: Nat Commun. 2022 Feb 3;13:655. doi: 10.1038/s41467-022-28280-1 (PMC8814036; doi:10.1038/s41467-022-28280-1)
Supplement: Supplementary file 4 — Description of Additional Supplementary Files [file 41467_2022_28280_MOESM4_ESM.pdf]

Description of Additional Supplementary Files

File Name: Supplementary Data 1
Description: Correlation of TMT protein with estimated protein and mRNA for (a) the TMT cohort, (b) the SRM cohort, and (c) the MSBB cohort. Pearson's correlation test was used for this comparison and the significance was adjusted with FDR for multiple comparisons.

File Name: Supplementary Data 2
Description: GO enriched for principal component of mRNA data. The enrichment analysis was conducted using one-sided Fisher's exact test. The significance levels were adjusted for multiple comparisons using FDR at 5%.

File Name: Supplementary Data 3
Description: Molecular characteristics defining protein predictability.

File Name: Supplementary Data 4
Description: Differential expression statistics of actual proteins, mRNAs and estimated proteins for (a) the TMT cohort, (b) the SRM cohort, and (c) the MSBB cohort.

File Name: Supplementary Data 5
Description: Differential expression of (a) proteins and (b) mRNAs for ADRD phenotypes. The value represents signed negative log10 of p-value.

File Name: Supplementary Data 6
Description: GO enrichment for protein-specific cognitive-decline associated genes. The enrichment analysis was conducted using one-sided Fisher's exact test. The significance levels were adjusted for multiple comparisons using FDR at 5%.

File Name: Supplementary Data 7
Description: Module assignment based on the estimated protein abundance.

File Name: Supplementary Data 8
Description: Protein module annotation based on gene ontology and cellspecific genes. The enrichment analysis was conducted using one-sided Fisher's exact test. The significance levels were adjusted for multiple comparisons using FDR at 5%.

File Name: Supplementary Data 9
Description: Comparison of TMT and SRM data in correlation and differential expression statistics.
